# circKCNQ5 promotes the proliferation of DNA-methyltransferase 3A R882 mutated acute myeloid leukemia cells by elevating high-mobility group box 1 expression

**DOI:** 10.1080/07853890.2025.2478309

**Published:** 2025-03-25

**Authors:** Yijian Chen, Xiaodan Zhu, Chuanming Lin, Rong Xu, Pengxiang Xu, Liuyan Xin, Lin Li, Liqun Zhang

**Affiliations:** ^a^ Department of Hematology, The First Affiliated Hospital of Gannan Medical University, Ganzhou, Jiangxi, P. R. China; ^b^ Department of Endocrinology, The First Affiliated Hospital of Gannan Medical University, Ganzhou, Jiangxi, P. R. China; ^c^ Quality Control Department, The First Affiliated Hospital of Gannan Medical University, Ganzhou, Jiangxi, P. R. China

**Keywords:** AML, proliferation, DNMT3A-R882 mutation, circKCNQ5, HMGB1

## Abstract

**Background:**

Patients with acute myeloid leukemia (AML) harboring the DNA-methyltransferase 3 A (DNMT3A) R882 mutation (DR882MUT) usually have a high recurrence rate and poor prognosis. circKCNQ5 levels were aberrantly elevated in patients with AML according to the microarray platform. Therefore, the purpose of this study is to investigate the effect and mechanism of circKCNQ5 on DR882MUT AML cell proliferation.

**Methods:**

A DR882MUT cell line model was established. circKCNQ5 expression in AML cells expressing wild-type DNMT3A (DNMT3A-WT) or DR882MUT was analyzed using RT-qPCR. The proliferation of DNMT3A-WT and DR882MUT AML cells after transfection was measured using a CCK-8 assay. High-mobility group box 1 (HMGB1) protein expression was assessed by western blotting. The regulatory mechanism of circKCNQ5 on HMGB1 expression was studied using RNA pull-down and co-immunoprecipitation assays.

**Results:**

circKCNQ5 expression increased gradually in HS-5, DNMT3A-WT, and DR882MUT AML cells. circKCNQ5 overexpression facilitated the proliferation of DNMT3A-WT KG-1a and HL-60 cells, whereas circKCNQ5 silencing blocked DR882MUT KG-1a and HL-60 cell proliferation. CircKCNQ5 interacts with HMGB1 and enhanced HMGB1 protein levels by inhibiting HMGB1 ubiquitination. HMGB1 protein levels increased gradually in HS-5, DNMT3A-WT, and DR882MUT AML cells. Furthermore, circKCNQ5 overexpression elevated HMGB1 protein levels in DNMT3A-WT KG-1a and HL-60 cells, whereas circKCNQ5 silencing reduced HMGB1 protein levels in DR882MUT KG-1a and HL-60 cells. HMGB1 overexpression remarkably increased the proliferative ability of DR882MUT KG-1a and HL-60 cells and circKCNQ5 silencing.

**Conclusions:**

These findings verified that circKCNQ5 promotes the proliferation of DR882MUT AML cells by increasing HMGB1 expression.

## Background

Acute myeloid leukemia (AML) is a malignant hematologic disease that is often accompanied by a rapid onset, poor prognosis, and relapse with drug resistance [1]. Chromatin regulatory gene changes with epigenetic modifications are associated with recurrent genetic abnormalities in AML [2]. DNA-methyltransferase 3 A (DNMT3A) is responsible for *de novo* methylation of CpG dinucleotides and is critical for establishing and maintaining cellular methylation patterns [3,4]. The DNMT3A mutation, a common genetic aberration, is present in approximately 30% of AML cases and can lead to ancestral or basal preleukemia cloning [5]. Similar to other lost-function mutations, the DNMT3A R882 mutation (DR882MUT) decreased methyltransferase activity on CpG substrates *in vitro* [6]. Recent evidence has claimed that DR882MUT is involved in multiple mechanisms of action and is associated with incomplete response rates, poor clinical outcomes, and recurrence in patients with AML [7]. However, the regulatory mechanisms of the downstream targets in AML with DR882MUT remain unclear. Therefore, it is crucial to identify the vulnerability of this cell population and explore it as a new therapeutic method for treating these patients.

Non coding RNAs, including circular RNAs (circRNAs), long noncoding RNA, and microRNA, play important roles in the development of AML [8,9]. circRNAs are conserved closed-ring single-stranded non-coding RNA produced by the back splicing of pre-mRNA transcripts [10]. Mounting evidence indicates that abnormally expressed circRNAs are potential biomarkers that play critical roles in AML tumorigenesis through multiple mechanisms [11]. For instance, circ_0004277 functions as a tumor-suppressing factor and is expressed at low levels in AML; circ_0004277 overexpression can block AML cell viability and metastasis [12]. circPTK2 levels were increased in AML plasma samples with cells, circPTK2 overexpression blocked AML cell proliferation with glycolysis and accelerated apoptosis with cell cycle arrest [13]. circKCNQ5 (hsa_circ_0004136) is a novel circRNA; however, little is known about its expression or function in cancer progression. Li et al. showed that circKCNQ5 is aberrantly expressed in multiple myeloma and regulates malignant behavior *via* glycolysis [14]. In AML, circKCNQ5 levels are aberrantly elevated in patients with AML according to the microarray platform [15,16]. circKCNQ5 could accelerate the malignant functions of AML cells [16,17]. However, the regulatory effect of circKCNQ5 on the malignant behavior of DR882MUT AML cells remains unknown.

In this study, AML cell lines overexpressing DR882MUT were constructed. Next, circKCNQ5 expression with function in DR882MUT AML cell lines was analyzed. Finally, we investigated the possible molecular mechanism by which circKCNQ5 modulates the proliferation and differentiation of DR882MUT AML cells. This study provides new theoretical evidence and potential therapeutic targets for DR882MUT AML patients, which may contribute to the development of novel targets and research strategies in clinical practice.

## Methods

### Cell culture, establishing DR882MUT cell lines, and transfection

Human normal mononuclear cell line HS-5 and human AML cell lines (KG-1a and HL-60) were obtained from the Shanghai boke Biotechnology (Shanghai, China). The cells were cultured in RPMI-1640 medium with 10% FBS and then incubated at 37 °C under 5% CO_2_. Small interfering RNAs (siRNAs) against circKCNQ5 (si-circKCNQ5), si-negative control (si-NC), overexpression circKCNQ5 plasmids (ov-circKCNQ5), overexpression HMGB1 plasmids (ov-HMGB1), and empty pcDNA3.1 plasmids (NC) were obtained from GenePharma (Shanghai, China). Cell transfection was performed using Lipofectamine 2000 Reagent following the manufacturer’s instructions (Invitrogen, Waltham, MA, USA). The DR882MUT cell line model was established as previously described [7]. Wild-type DNMT3A (DNMT3A-WT) or DR882MUT (R882H, base 2645 G > A) were synthetized by GenePharma (Shanghai, China) and inserted into lentiviral vector plvx-pgk-zsgreen-puro. Then the lentiviral plasmid and packaging plasmid were transfected into 293 T cells together. After culture 3 days, the lentiviruses in cell culture supernatant were collected and concentrate it for storage. Then stably expressing cell lines were selected using puromycin. Briefly, 5 × 10^4^ KG-1a and HL-60 per well were plated into a 24-well plate and infected with the above lentiviruses for 6h. Cell in the control group was not transfected with lentivirus. Then cells were treated with 5 μg/ml puromycin. After cells in the control group were completely dead, the cells which infected DNMT3A-WT and DR882MUT lentiviral vector were expanded culture, and maintained culture in 1 μg/ml puromycin for 4 weeks to obtain stable transformed strains. Then cells were collected for sequencing identification (Sangon, Shanghai, China) to confirm the success of DR882MUT mutation. Successfully mutated cells were further cultured for 3 generations and stored for future use.

### RT-qPCR

Total RNA from cells was isolated using TRIzol (Invitrogen) and treated with RNase R (Sigma-Aldrich, St. Louis, MO, USA). Next, cDNA was reverse transcribed using 1 μg RNA. qPCR was performed using an ABI PCR system (Applied Biosystems, Foster City, CA, USA). Fold changes in the transcripts were computed applying the 2^−ΔΔCT^ method, and GAPDH was used as an internal reference. The circKCNQ5 primer sequences used were as follows: forward: 5′-CATTCGAATCTGGTCTGCGG-3′, reverse: 5′ -TGTGTGCTCAGGGATGGTAG-3′; high-mobility group box 1 (HMGB1) forward: 5′-TGCTCTGAGTATCGCCCAAA-3′, reverse: 5′ -GCAGCCTTCTTTTCATAAGG-3′; GAPDH forward: 5′- CCCCCATGTTCGTCATGGGT-3′, reverse: 5′ - TCATGGATGACCTTGGCCAG −3′.

### Cell proliferation and cycle assays

Cell proliferation was assessed using the Cell Counting Kit-8 assay (beyotime, Shanghai, China). After transfection, cells were seeded into 96-well plates. Subsequently, the cells were incubated for 0, 24, 48, and 72 h. Subsequently, 10 µL of CCK-8 solution was added to every well at corresponding points of time, and placed at 37 °C after 2 h. The OD of the cells in each well was measured at 450 nm by using a microplate reader. Cell cycle assays were conducted using a Cell Cycle Detection Kit (KeyGen, Jiangsu, China). After culture 24h, cells were collected and wash once with PBS, then centrifuged and adjusted the cell concentration to 1 × 10^6^/ml; Then 1 ml cell suspension was centrifuged to remove the supernatant and added 500 μl of 70% cold ethanol to the cells for fixing for 2 h. Next, cells were centrifuged to remove the supernatant and washed with PBS; Finally, cells were centrifuged to remove the supernatant, added 500 μl of PI/RNase A staining solution to incubate at 25 °C in the dark for 30 min, and detected using flow cytometry (BD, San Jose, CA,USA).

### RNA pull-down, RNA immunoprecipitation, and co-immunoprecipitation assays

PureBinding^®^RNA-Protein pull-down Kit (Geneseed, Shanghai, China) was used for RNA pull-down. Briefly, DR882MUT- KG-1a were collected and lysed in Capture Buffer and centrifuged at 12000 × *g* for 10 min at 4 °C and collected the supernatant. Then, 50 μl supernatant were retained as the input sample. Next, biotinylated circKCNQ5 sense and antisense was incubated with streptavidin magnetic beads for 10 min at 4 °C; then added 450 μl supernatant for 4 h at 4 °C. The beads were rinsed twice with pre-cooled cracking buffer followed by three rinses with PBS. Subsequently, the immunoprecipitation complex was isolated and purified, and enrichment of HMGB1 was assessed by western blotting. RNA immunoprecipitation (RIP) was performed using an PureBinding^®^RNA Immunoprecipitation Kit (Geneseed). Briefly, the cells were then collected and lysed in lysis buffer and centrifuged at 12000 × *g* for 10 min at 4 °C and collected the supernatant. Then, 100 μl supernatant were retained as the input sample. Next, Protein A/G Magnetic Beads were added 5 μg anti-HMGB1 and IgG for incubating at 4 °C for 1 h. Then 400 μl supernatant was added and incubated for 2 h at 4 °C. After the beads were washed and eluted with elution buffer, RNA was collected, treated with RNase R, and analyzed using RT-qPCR. HMGB1 ubiquitin was analyzed by PureBinding^®^Co-Immunoprecipitation (Co-IP) Kit (Geneseed). Briefly, Protein A/G Magnetic Beads were added 5 μg anti-HMGB1 and IgG for incubating at 4 °C for 1 h. Then 400 μl total protein was added and incubated for 2 h at 4 °C. After the beads were washed and collected protein for perform ubiquitination analysis using western blotting.

### Western blotting

A Protein Extraction Kit (Sangon Biotech, Shanghai, China) was used to harvest the total protein from the cells. Equal amounts of protein lysate were separated by SDS-PAGE and transferred onto PVDF membranes (Millipore, Burlington, MA, USA). After blocking for non-specific binding, the membrane was incubated with the following primary antibodies at 4 °C overnight: anti-HMGB1 (1:800; ab18256; Abcam, Cambridge, UK), anti-ubiquitin (1:1000; ab134953; Abcam), and anti-GAPDH (1:1500; ab181602; Abcam). After rinsing, the membranes were incubated with HRP-labeled antibody (1:1800; ab205718; Abcam) for 2 h at 25 °C and then washed. Finally, the proteins were quantified using enhanced chemiluminescence (Keygentec, Nanjing, China) and ChemiDoc^™^ XRS systems (Bio-Rad, Hercules, CA, USA).

### Statistical analysis

Statistical analysis was performed using IBM SPSS Statistics for Windows, version 22.0. Data are presented as means ± standard deviation (SD). Differences between two or more groups were compared using the *t*-test or one-way ANOVA. *P*-values < 0.05 were considered statistically significant.

## Results

### circKCNQ5 expression was enhanced in DNMT3A-MUT AML cells

**Figure 1.**
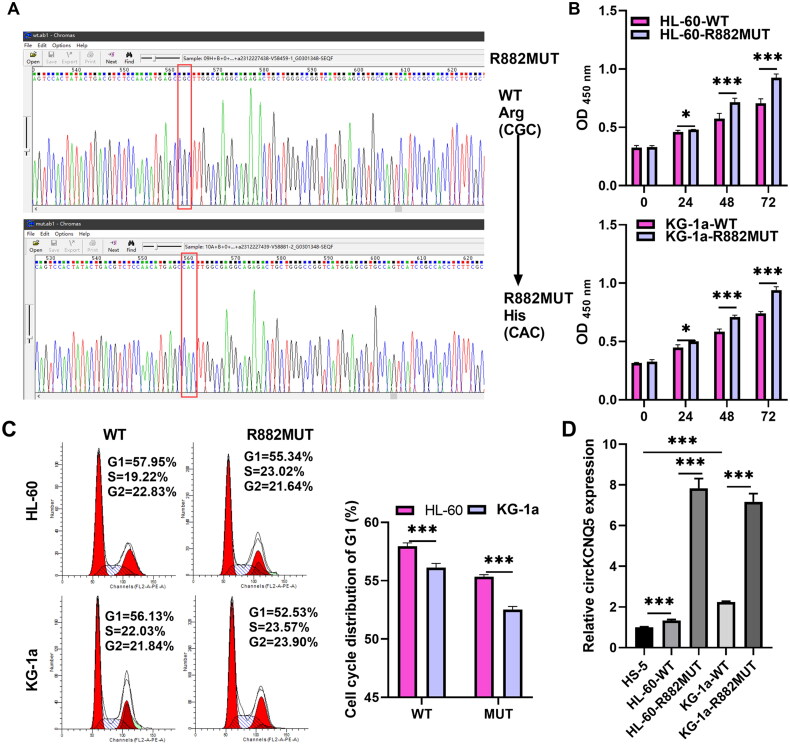
circKCNQ5 Expression was enhanced in DNMT3A-MUT AML cells. (A) Sequencing results confirmed successful construction of DNMT3A-R882MUT (DR882MUT) AML cells. (B and C) Cell proliferation and cell cycle of DNMT3A-WT and DR882MUT KG-1a and HL-60 cells were measured using CCK-8 assay and flow cytometry. (D) circKCNQ5 expression levels in HS-5 cells, DNMT3A-WT, and DR882MUT AML cells were measured using qRT-PCR. (*p < 0.05 and ***p < 0.001).

The sequencing results showed that the mutagenesis process was successful (Figure 1A). In addition, the cell proliferation and cell cycle assay results indicated that proliferation was increased; the G1-phase was reduced in DR882MUT AML cells compared to DNMT3A-WT AML cells, suggesting that DR882MUT promoted proliferation and the development of the G1-phase toward the S-phase (Figure 1B and C). To observe whether circKCNQ5 was abnormally expressed in AML cells, circKCNQ5 expression levels in HS-5 cells, DNMT3A-WT, or DR882MUT AML cells were first identified. RT-qPCR analysis showed that circKCNQ5 expression was remarkably elevated in DNMT3A-WT and DR882MUT AML cells compared to mononuclear HS-5 cells, whereas circKCNQ5 expression in DR882MUT KG-1a and HL-60 cells was higher than that in DNMT3A-WT KG-1a and HL-60 cells (Figure 1D).

### circKCNQ5 overexpression enhanced proliferation of DNMT3A-WT AML cells

**Figure 2.**
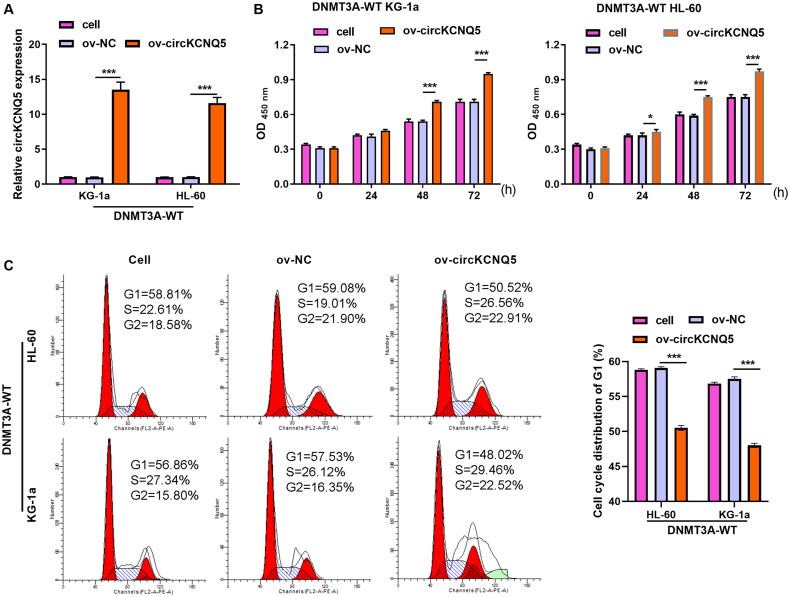
circKCNQ5 Overexpression facilitated proliferation of DNMT3A-WT AML cells. (A) circKCNQ5 expression in DNMT3A-WT KG-1a and HL-60 cells after si-circKCNQ5 or si-NC transfection was measured using qRT-PCR. (B and C) Cell proliferation and cell cycle of DNMT3A-WT KG-1a and HL-60 cells after ov-circKCNQ5 or ov-NC transfection were assessed using CCK-8 assay and flow cytometry. (***p < 0.001).

To explore the functional effects of circKCNQ5 on DNMT3A-WT KG-1a and HL-60 cells, the cells were transfected with ov-circKCNQ5 or ov-NC. RT-qPCR results showed that circKCNQ5 expression levels were increased in the ov-circKCNQ5 group compared to in the ov-NC group (Figure 2A). Functionally, the cell proliferation and cell cycle assay results indicated that circKCNQ5 overexpression evidently facilitated proliferation and promoted the development of the G1-phase toward the S-phase in DNMT3A-WT KG-1a and HL-60 cells compared to transfected ov-NC (Figure 2B and ^C^).

### circKCNQ5 silencing prevented DR882MUT AML cell proliferation

**Figure 3.**
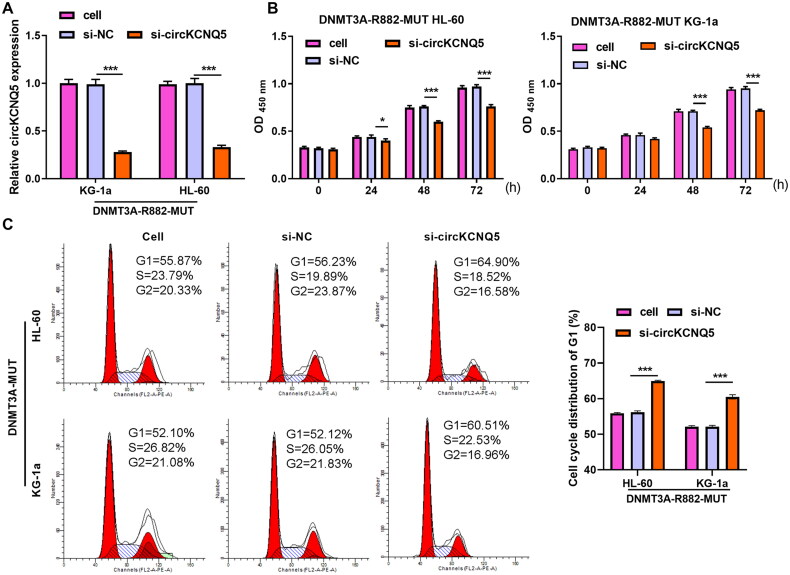
circKCNQ5 Silencing blocked DR882MUT AML cell proliferation. (A) circKCNQ5 expression levels in DR882MUT KG-1a and HL-60 cells after si-circKCNQ5 or si-NC transfection were measured using qRT-PCR. (B and C) Cell proliferation and cell cycle of DR882MUT KG-1a and HL-60 cells after transfection with si-circKCNQ5 or si-NC were assessed using CCK-8 assay and flow cytometry. (***p < 0.001).

DR882MUT KG-1a and HL-60 cells were transfected with si-circKCNQ5 or si-NC to investigate the functional effects of circKCNQ5. The data suggested that circKCNQ5 expression was both markedly reduced in DR882MUT KG-1a and HL-60 cells after transfection with si-circKCNQ5 (Figure 3A). Functionally, the proliferation and cell cycle of DR882MUT KG-1a and HL-60 cells were blocked by silencing circKCNQ5 (Figure 3B and C).

### circKCNQ5 silencing suppressed HMGB1 protein expression

**Figure 4.**
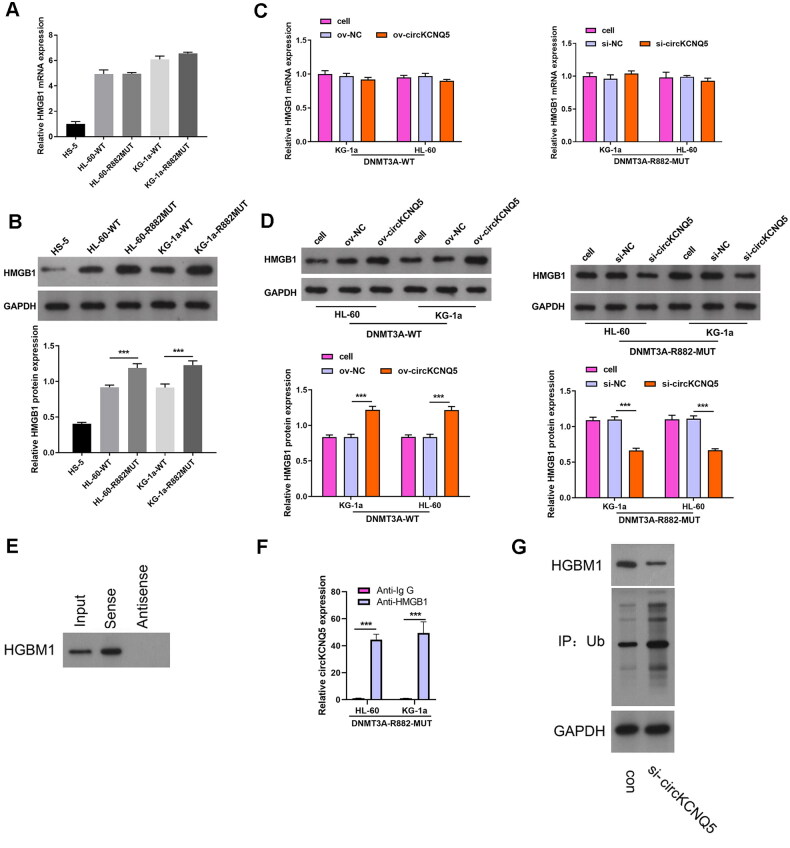
circKCNQ5 Silencing suppressed HMGB1 protein expression. (A) HMGB1 mRNA expression in HS-5 cells, and DNMT3A-WT and DR882MUT AML cells was measured by qRT-PCR. (B) HMGB1 protein levels in HS-5 cells, and DNMT3A-WT and DR882MUT AML cells was measured by Western blotting. (C) HMGB1 mRNA expression in DNMT3A-WT KG-1a and HL-60 cells after transfection with ov-circKCNQ5 or ov-NC and in DR882MUT KG-1a and HL-60 cells after transfection with si-circKCNQ5 or si-NC was measured by qRT-PCR. (D) MGB1 protein levels in DNMT3A-WT KG-1a and HL-60 cells after transfection with ov-circKCNQ5 or ov-NC were measured by Western blotting. HMGB1 protein levels in DR882MUT KG-1a and HL-60 cells after transfection with si-circKCNQ5 or si-NC was measured by using Western blotting. (E) Combination of circKCNQ5 and HMGB1 was detected using RNA pull-down assay and by Western blotting. (F) Combination of circKCNQ5 and HMGB1 was detected using RIP assay and RT-qPCR. (G) HMGB1 ubiquitin and expression in circKCNQ5 silencing cells was measured using Co-IP assay and by Western blotting. (***p < 0.001).

A previous study found that HMGB1 is related to DR882MUT [18]. In addition, our previous study showed that HMGB1 is overexpressed in AML and is associated with its occurrence and progression [19]. HMGB1 mRNA and protein levels were significantly increased in wild-type (DNMT3A-WT AML cells) and mutant cells (DR882MUT AML cells), compared with HS-5 cells (Figure 4A and ^B^). In addition, HMGB1 protein levels were significantly enhanced in DNMT3A-WT AML cells after circKCNQ5 overexpression and significantly reduced in DNMT3A-MUT AML cells after circKCNQ5 downexpression, whereas HMGB1 mRNA had no significant change (Figure 4C and ^D^). The results indicate that circKCNQ5 may regulate the degradation of HMGB1 through protein post-translational modifications. Next, to decipher the possible interaction between circKCNQ5 and HMGB1 in regulating AML cell proliferation, RNA pull-down and RIP showed that circKCNQ5 interacted with HMGB1 to form a complex (Figure 4E and F). Additionally, circKCNQ5 silencing promoted HMGB1 ubiquitination and reduced HMGB1 protein levels (Figure 4G).

### HMGB1 overexpression could turnaround repressive effect of circKCNQ5 silencing on DR882MUT AML cell proliferation

**Figure 5.**
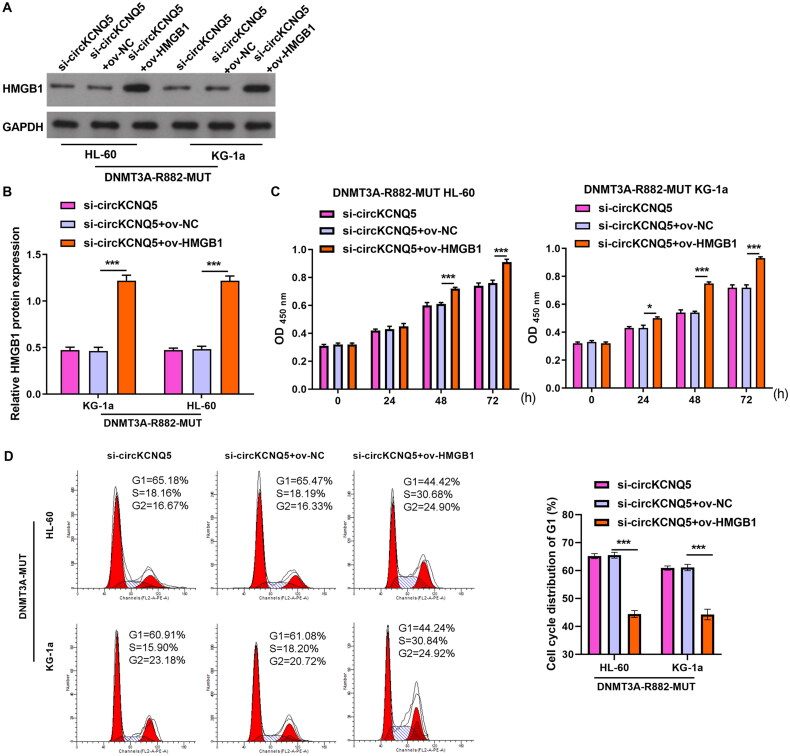
HMGB1 Overexpression promoted proliferation abilities of DR882MUT KG-1a and HL-60 cells. (A and B) HMGB1 protein levels in DR882MUT KG-1a and HL-60 cells after co-transfection with ov-HMGB1 and si-circKCNQ5 were measured by Western blotting. (C and D) Proliferation and cell cycle of DR882MUT KG-1a and HL-60 cells after co-transfection with ov-HMGB1 and si-circKCNQ5 were analyzed using CCK-8 assay and flow cytometry. (***p < 0.001).

Next, to explore whether circKCNQ5 exerted its function in DR882MUT AML cells through HMGB1, a rescue experiment was performed by cotransfecting ov-HMGB1 and si-circKCNQ5 into DR882MUT KG-1a and HL-60 cells. HMGB1 protein levels were remarkably elevated in both DR882MUT KG-1a and HL-60 cells after co-transfection with ov-HMGB1 and si-circKCNQ5 (Figure 5A and ^B^). HMGB1 overexpression remarkably increased the proliferative ability and promoted the development of G1 toward the S-phase of DR882MUT KG-1a and HL-60 cells after co-transfection with ov-HMGB1 and si-circKCNQ5 (Figure 5C and ^D^).

### HMGB1 downexpression could turnaround repressive effects of circKCNQ5 overexpression on DNMT3A-WT AML cell proliferation

**Figure 6.**
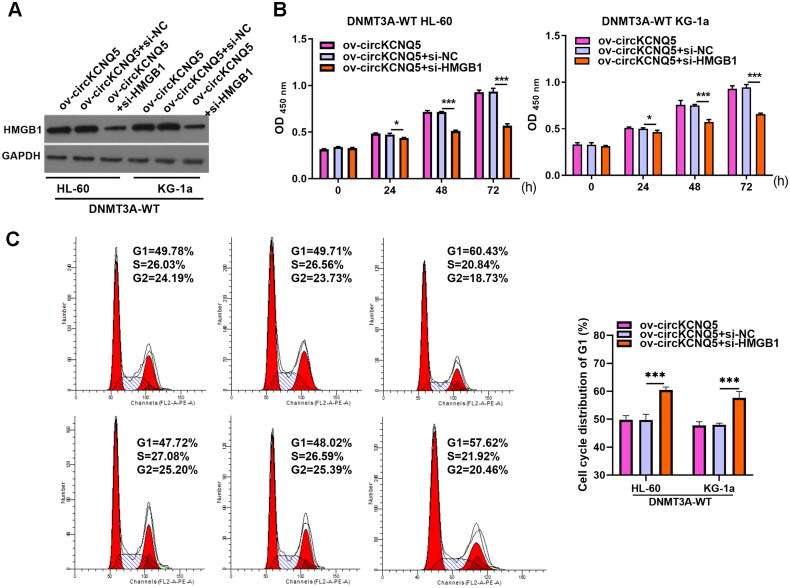
HMGB1 Downexpression inhibited proliferation abilities of DNMT3A-WT KG-1a and HL-60 cells. (A) HMGB1 protein levels in DNMT3A-WT KG-1a and HL-60 cells after co-transfection with si-HMGB1 and ov-circKCNQ5 were measured by Western blotting. (B and C) Proliferation and cycle of DNMT3A-WT KG-1a and HL-60 cells after co-transfecting si-HMGB1 and ov-circKCNQ5 were analyzed using CCK-8 assay and flow cytometry. (*p < 0.05 and ***p < 0.001).

Next, to explore whether circKCNQ5 exerted its function in DNMT3A-WT AML cells through HMGB1, a rescue experiment was performed by cotransfecting si-HMGB1 and ov-circKCNQ5 into DNMT3A-WT KG-1a and HL-60 cells. HMGB1 protein expression levels were remarkably reduced in both DR882MUT KG-1a and HL-60 cells after co-transfection with si-HMGB1 and ov-circKCNQ5 (Figure 6A). HMGB1 downregulation markedly inhibited proliferation and reduced the development of G1 toward the S-phase of DNMT3A-WT KG-1a and HL-60 cells after co-transfection with si-HMGB1 and ov-circKCNQ5 (Figure 6B and ^C^).

## Discussion

DNMT3A mutations are frequently found in AML, and more than half of these mutations occur in arginine 882 [20]. Thus, it is vital to explore the characteristics of this cell population and discover and exploit their weaknesses in DR882MUT AML therapy. Here, we confirmed that circKCNQ5 and HMGB1 levels were elevated in both DNMT3A-WT and DR882MUT AML cells. CircKCNQ5 overexpression facilitated the proliferation of DNMT3A-WT AML cells, whereas circKCNQ5 silencing prominently blocked DR882MUT AML cell proliferation. Mechanistic analysis revealed that circKCNQ5 silencing suppressed HMGB1 protein expression and facilitated degradation. circKCNQ5 overexpression elevated HMGB1 protein levels. Notably, HMGB1 overexpression repressed the effect of circKCNQ5 silencing on DR882MUT AML cell proliferation. These findings highlighted circKCNQ5 may be a promising target for DR882MUT AML treatment and provide a novel functional mechanism for circKCNQ5 in DR882MUT AML pathological progression.

Recently, numerous studies have reported that multiple circRNAs are aberrantly expressed in AML and participate in the regulation of AML occurrence and development [21]. Similar to Wang et al. [22], circ_0040823 expression was downregulated in leukemia cells and patients with AML, and circ_0040823 overexpression blocked AML cell proliferation and cell cycle progression, whereas facilitating apoptosis and cell cycle arrest. circ-PTK2 is overexpressed in patients with AML, circ-PTK2 knockdown blocked AML cell proliferation and triggered apoptosis [23]. circKCNQ5 is the parental gene of circ_0004136 and is located on chr6. However, little information is available on the role of circKCNQ5 in cancer. In multiple myeloma, the circKCNQ5 expression was elevated in multiple myeloma tissues and cells, circKCNQ5 silencing impeded multiple myeloma cells proliferation, migration, invasion, and glycolysis, suggesting circKCNQ5 contributed to multiple myeloma tumorigenesis and development [14]. In AML, Hu et al. [24] revealed that circKCNQ5 was aberrantly elevated in AML cells and bone marrow samples from patients with AML, and that circ_KCNQ5 silencing largely impeded AML cell proliferation and accelerated apoptosis by promoting RAB10 expression by sponging miR-622. Similarly, the present study revealed aberrantly enhanced circKCNQ5 in DR882MUT AML cells; circKCNQ5 overexpression facilitated the proliferation of DNMT3A-WT AML cells, whereas circKCNQ5 silencing prominently blocked DR882MUT AML cell proliferation. These data suggested that circKCNQ5 knockdown restricted the progression of DR882MUT AML cells by blocking cell proliferation.

In the current study, circKCNQ5 was shown to interact with HMGB1 and enhance HMGB1 protein expression by inhibiting HMGB1 ubiquitination. Ubiquitination of HMGB1 leads to its degradation [25,26]. HMGB1 is a highly conserved non-histone nuclear protein that serves as a chromatin-binding factor that bends DNA and facilitates the acquisition of transcription protein components from specific DNA targets [27]. As a multifunctional protein, HMGB1 has been widely investigated [28]. HMGB1 plays an important role in the pathogenesis of leukemia, and its upregulation is closely related to chemotherapy resistance by regulating autophagy [29]. Liu et al. [30] revealed that HMGB1 is highly expressed in patients with AML and is involved in the occurrence and development of AML by inhibiting apoptosis, promoting proliferation, and blocking myeloid differentiation of AML cells, indicating that HMGB1 is a vital regulatory factor for myeloid differentiation and leukemogenesis in AML. In the present study, HMGB1 protein levels were markedly elevated in DNMT3A-WT and DR882MUT AML cells. circKCNQ5 overexpression elevated HMGB1 protein levels in DNMT3A-WT AML cells, whereas circKCNQ5 silencing reduced HMGB1 protein levels in DR882MUT AML cells. The functional assay revealed that HMGB1 overexpression markedly increased the proliferative abilities of DR882MUT AML and circKCNQ5 silencing, whereas HMGB1 downregulation remarkably inhibited the proliferative abilities of DNMT3A-WT AML and circKCNQ5 overexpression. These results further confirmed that circKCNQ5 promotes the proliferation of DR882MUT AML cells by increasing HMGB1 expression.

There are several limitations to this study. First, it is unclear if circKCNQ5 has other regulatory targets besides HMGB1. The downstream pathway of HMGB1 has not been studied in DR882MUT AML cells. Additionally, the function of circKCNQ5 has not been investigated *in vivo*. Lastly, the expression of circKCNQ5 in DR882MUT AML patients remains unclear. Therefore, future studies will aim to solve these limitations and improve upon the current findings.

In summary, circKCNQ5 and HMGB1 were dramatically elevated in DR882MUT AML cells, and circKCNQ5 promotes the proliferation of DR882MUT AML cells by increasing HMGB1 expression. Thus, circKCNQ5 is a promising biomarker and treatment target for DR882MUT AML. This study has the potential to contribute to the development of new targets and treatment strategies for DR882MUT AML patients in clinical practice.

## Data Availability

The datasets used and/or analyzed in the current study are available from the corresponding author upon reasonable request.
